# Pragmatics as Metacognitive Control

**DOI:** 10.3389/fpsyg.2015.02057

**Published:** 2016-01-14

**Authors:** Mikhail Kissine

**Affiliations:** Autisme en Contexte: Théorie et Expérience, Université libre de BruxellesBruxelles, Belgium

**Keywords:** pragmatic process, metacognition, Theory of Mind, autism spectrum disorder, indirect speech act, irony, implicature, Relevance theory

## Abstract

The term “pragmatics” is often used to refer without distinction, on one hand, to the contextual selection of interpretation norms and, on the other hand, to the context-sensitive processes guided by these norms. Pragmatics in the first acception depends on language-independent contextual factors that can, but need not, involve Theory of Mind; in the second acception, pragmatics is a language-specific metacognitive process, which may unfold at an unconscious level without involving any mental state (meta-)representation. Distinguishing between these two kinds of ways context drives the interpretation of communicative stimuli helps dissolve the dispute between proponents of an entirely Gricean pragmatics and those who claim that some pragmatic processes do not depend on mind-reading capacities. According to the model defended in this paper, the typology of pragmatic processes is not entirely determined by a hierarchy of meanings, but by contextually set norms of interpretation.

## 1. Introduction

Everyone agrees that, at some level or another, utterance interpretation involves integrating contextual information to fill in the gap between the encoded linguistic meaning and what is actually communicated. To facilitate the discussion to come, context-dependent contents may be subsumed under two categories. First, context is needed to determine the meaning literally conveyed by the utterance (e.g., Bach, 1994; Carston, 2002; Recanati, 2004). Let us call such cases *primary meanings*:

(1) I have already had breakfast. [meaning that the speaker had breakfast on the day of the utterance]
(2) The fridge is empty. [meaning that the fridge does not contain anything suitable for a proper meal]
(3) You're not going to die. [said to child crying because of a minor cut, meaning that she is not going to die from that cut]
(4) Peter left Mary and she started to drink. [meaning that Mary started to drink after and because Peter left her]

Second, context is of course needed to recover meanings that are clearly different from or independent of the utterance literal content. Let us call context-dependent interpretations of this sort *secondary meanings*. Standard examples of secondary meanings include:

(5) *Irony*: This is the best movie I ever saw. [meaning that the speaker really hated it]
(6) *(Particularized conversational) implicatures*:
  - a. The candidate's command of English is excellent and his attendance to tutorials regular. [in a letter of recommendation for a lectureship in philosophy, strongly suggesting that the candidate is not suitable for the position];
  - b. There is a garage round the corner. [to someone who is out of petrol, conveying that this garage should be open and selling petrol] (Grice, 1975)
(7) *Indirect speech acts*: It is cold in here. [meant as a request to close the window]

The list of pragmatic phenomena just introduced is by no means exhaustive; there are many other aspects in which context influences utterance interpretation. For instance, I leave aside here the much discussed issue of “generalized” implicatures (e.g., Noveck, 2001; Geurts, 2010). Furthermore, the main claim of the paper is precisely that from a processing point of view neither secondary nor primary meanings constitute a natural class. Nonetheless, this two fold distinction is useful to introduce a chief theoretical divergence within the field of cognitively oriented pragmatics. The first major view stems from an adoption of Grice's (1957) rational reconstructions into a psychological theory of interpretation.

Proponents of this position claim that any kind of pragmatic processing involves inferring the intentions that underlie the speaker's communicative behavior. They see pragmatic processing, associated with the derivation of both primary or secondary meanings, as a homogenous cognitive capacity, inherently rooted within Theory of Mind (understood as the capacity to attribute and reason about mental states).

The second camp accepts that Gricean inferences about communicative intentions are needed to reach secondary meanings but holds that the derivation of primary meanings is underpinned by accessibility-based, non-inferential processes. Under this view, the derivation of primary meanings would then involve Theory of Mind-independent pragmatic processing.

This debate raises crucial issues about the relationship between pragmatics and Theory of Mind, and about the (alleged) modularity of pragmatic processing. But instead of taking camps, this paper advocates a change of perspective. The term “pragmatics” is often used to refer without distinction, one the one hand, to the contextual choice of norms of interpretation and, on the other hand, to the context-sensitive processes guided by these interpretative norms. I will argue that pragmatics in the first sense depends on language-independent contextual factors that can, but need not, involve Theory of Mind; in the second sense, pragmatics is a language-specific metacognitive process, which may unfold at an unconscious level without involving any kind of meta-representation.

This paper is organized as follows. In the next two sections I will outline the main features of the two conflicting visions of pragmatic processing: monolithic, post-Gricean inferential accounts and more heterogenous, accessibility-based approaches. In Section 2, I will take Relevance theory as a paradigmatic example of the former kind of analysis (Sperber and Wilson, 1995, 2002), and, in Section 3, the distinction between primary and secondary pragmatic processes, advocated by Recanati (2004), as an example of the latter. By no means should this choice be taken as a limitation of the argument scope to these two particular theories. Simply, while many authors leave implicit the workings of the cognitive model they adhere to, both Sperber and Wilson, and Recanati provide starkly articulated descriptions of their cognitive commitments. From the critical discussion of these two polar positions, I will argue that a psychologically valid pragmatic model should distinguish, as independent dimensions, between types of meanings (primary vs. secondary), and types of pragmatic processes (accessibility-based vs. Gricean). I will then outline of model that would meet such a constraint. The solution I propose is based on the two-tiered theory of epistemic acceptance, which I borrow from Proust (2013, pp. 169–184) and summarize in Section 4. Proust's insight is that one should not confuse the choice or acceptance of an epistemic norm with the acceptance of a level of epistemic success relative to this norm. Transposing this idea to pragmatics, I will suggest, in Section 5, that one should distinguish between, one hand, between contextually determined norms of interpretation and, on the other hand, cognitive processes that control and lead to the achievement of this interpretative goal. The change of perspective advocated in this paper naturally accommodates experimental data that indicate that pragmatic processing is possible without sophisticated Theory of Mind, and opens interesting perspectives on the interpretation of experimental results in pragmatics.

## 2. Relevance theory

Grice's long-lasting insight is that (non-natural) meaning can be rationally reconstructed in terms of complex communicative intentions (Grice, 1957). Under such a reconstruction, a speaker S (non-naturally) means that *p* if, and only if:

- S has the intention *i*_1_ to make the addressee believe that *p*;
- S has the intention *i*_2_ that the recognition of *i*_1_ by the addressee be a reason for him to believe that *p*.

This rational reconstruction of communicative behavior has been quickly transposed into a psychological view of how utterance contents are recovered by addressees, and became deeply entrenched in experimental psychology and cognitive science. To date, the most fully articulated version of such a post-Gricean approach remains Relevance theory (Sperber and Wilson, 1995).

### 2.1. Classic relevance theory: a pragmatic module

According to Sperber and Wilson, communicative stimuli activate a specific interpretation process. While non-communicative intentional behavior is interpreted by attributing to the agent an intention to act, according to them the interpretation of communicative behaviors is mediated by the attribution an *informative intention*. Informative intentions are intentions to provide the addressee with (dispositions to acquire) new beliefs or to reinforce existing ones. To exemplify Sperber and Wilson's distinction between intentions to act and informative intentions, think first of a stranger on the bus scratching her head. This is an instance of a non-communicative gesture; the stranger's behavior will be interpreted as resulting from an intention to relieve itching. By contrast, imagine next, that, when asked about my opinion about a particularly difficult paper, I demonstratively scratch my forehead. Here my gesture is communicative; according to Sperber and Wilson, it will be interpreted by inferring a certain informative intention from my behavior, e.g., an intention to make my addressee acquire or reinforce the belief that I do not have a ready-made answer.

Communicative stimuli, linguistic, and non-linguistic alike, can be associated with a virtually infinite number of informative intentions. The act of scratching my forehead could for instance mean that I find the answer difficult, but also that I do not feel comfortable with answering your question because I am personally acquainted with the author of the paper. Or, to take a linguistic example, an utterance of *I can't drink* may mean that I cannot drink alcohol because I am driving, that I do not want to have alcohol because I am often agressive when inebriated, that I have already had too much alcohol, that I cannot ingest any liquid because I have a blood test in an hour, etc. Relevance theory explains how the range of possible interpretations gets narrowed down by appealing to (the relatively uncontroversial) hypothesis that human cognition is geared toward an optimal balance between the cognitive effects of processing and the processing efforts required to reach these effects. In the case of communication, the quantity of the effects of an utterance can be modeled as the number of new practical and theoretical implications allowed by the output of its interpretation. Communicative behaviors, according to Sperber and Wilson, are always perceived as worth processing: to use their terms, communicative stimuli come “with their own presumption of relevance.”

The simplest interpretative procedure would be, then, to infer from the communicative stimuli the informative intention that is the most relevant from one's own point of view. Sperber (1994) suggests that this strategy, which he dubs “Naive Optimism,” is used by young children. Now, what is relevant from one's own, egocentric point of view may be different from the meaning the speaker actually intended to communicate. The core of the Gricean conception of speaker's meaning is that it should be overt; a speaker usually intends that her addressee recognizes her informative intention. Exploiting this idea, Relevance theory posits that the optimal way to reach communicative success, called “Sophisticated Understanding” by Sperber, is to attribute to the speaker the informative intention this speaker is likely to have intended to make mutually manifest to her and to her addressee. That is, one should base one's interpretation on attributing to the speaker a *communicative intention* to make mutually manifest an informative intention. The interpretative inference then runs from the communicative stimuli to the communicative intention that is the most relevant, given the speaker's abilities and preferences, to the informative intention embedded within this communicative intention.

Importantly, while Relevance theorists admit the existence of different interpretative strategies, with varying levels of complexity, they hold that the output of any kind of interpretative process—be it Naive Optimism or “Sophisticated Understanding”—involves the attribution of an informative intention to the speaker. It is this assumption that compels Sperber and Wilson to posit the existence of a unitary pragmatic module.

To see why, recall that an informative intention is a mental state whose content includes the representation of mental states (speaker's beliefs). In spite of recent evidence of early first-order Theory of Mind (Onishi and Baillargeon, 2005; Baillargeon et al., 2010), there is a consensus that children are not capable to attribute such complex, second-order mental states until the age of seven (Perner and Wimmer, 1985; Leekam and Prior, 1994). And yet, very young children are apt conversationalists, who prove to be sensitive to the context of the conversation and to the interlocutor's perspective. To give a few examples, infants display pointing behavior with a clearly informative function, which is, moreover, constrained by their social partner's state of knowledge (Liszkowski et al., 2006, 2008). They also interpret ambiguous requests relative to their partner's needs and intentions (Grosse et al., 2010; Schulze et al., 2013). Around thirty months, children attempt to correct an adult who misunderstood their request even though they are handed the requested object (Shwe and Markman, 1997). Three-year-olds also display sensitiveness to the speaker's perspective in reacting to synonymous labels; they are puzzled when their conversational partner suddenly shifts from using one name for an object to another, synonymous one, but not when a new speaker, who did not participate in the ongoing exchange, uses this synonym (Matthews et al., 2010).

In brief, there is a robust set of developmental data showing that, from a very young age, children use contextual cues to interpret and produce communicative behavior, even though they do not master second-order mental state attribution. In order to account for these empirical facts, Sperber and Wilson (2002) propose that pragmatic processing is underpinned by a specific cognitive module, devoted to the interpretation of communicative behavior. This pragmatic module would be rooted within a more general Theory of Mind, and would have an independent, and more precocious, developmental trajectory. Its output inevitably is the representation of the speaker's communicative or, at least, informative intention. Importantly, for Sperber and Wilson, this holds for the output of any interpretative process that goes beyond conventional, linguistically encoded meaning.

### 2.2. Implicatures: material vs. behavioral

As pointed out by Jary (2013), in Sperber and Wilson's model, the functioning of the pragmatic module itself does not necessarily involve the representations of the speaker's mental states. That is, utterance content is not necessarily recovered through inferences about speaker's intentions. Within the context of the conversation, the linguistic content of the utterance activates certain interpretations, the selection of these interpretations being warranted by the general expectation of relevance. Imagine a context where S is offered a coffee and a croissant and responds with an utterance of (8). This utterance makes accessible the contextually enriched primary meaning in (9). The topic of the conversation also makes accessible the background assumption in (10). The conjunction of (9) and (10) (non-monotonically) allows the conclusion in (11)—which corresponds to an implicature of the utterance in (8).

(8) I have already had breakfast.
(9) The speaker has already had breakfast today.
(10) Having had breakfast on a given day is a good reason for refusing a breakfast on that same day.
(11) The speaker does not want a coffee and a croissant.

Importantly, the derivation of the implicature in (11) does not have to chronologically follow that of the primary meaning (9). Rather, the principle of Relevance leads the interpreter to expect that the speaker's utterance will make manifest a range of additional consequences, viz. secondary meanings. Some such secondary meanings are made salient in the context; in the course of the interpretation process primary and secondary meanings are then adjusted, so that the primary meanings provide inferential warrant to the secondary ones. But, as we just saw, such an inference is possible without any reasoning about speaker's intentions taking place. [Even though Jary's (2013) argument for non-mentalistic derivation of material implicatures thus allows for a mutual adjustment between secondary and primary meanings, in still unpublished work (Jary, unpublished manuscript), he suggests that the derivation of primary meaning may not even be necessary.]

A similar rationale may be applied to indirect speech acts. Imagine a context where the window is open and the speaker utters (12). Provided that it is desirable for the addressee to relieve the speaker's unpleasant feeling of cold, the primary content (13) can combine with the assumption that closing windows makes the air warmer to lead to the decision to close the window. In other words, without the mediation of any hypotheses about speaker's intentions the utterance of (12) can serve as a reason to close the window, and thus lead to a contextually appropriate interpretation (Kissine, 2013, pp. 102–125).

(12) It is cold in here.
(13) The speaker is cold.

The secondary meaning (11), derived from (8), is an instance of what Jary calls *material implicature*. As we just saw, provided an overall expectation of Relevance, the derivation of material implicatures does not require hypotheses about the speaker's mental states. In this respect, material implicatures contrast with what Jary calls *behavioral implicatures*, whose derivation does require premises about the speaker's intentions, beliefs or desires. Take Grice's (1975) classic example of a recommendation letter which reads as (14). In order to derive the implicature that the candidate is not suitable for the position, in addition to the general presumption of cooperativeness, one needs premises such as (15) and (16).

(14) The candidate's command of English is excellent and his attendance to tutorials regular.
(15)The author of the letter knows that the candidate's competence in philosophy is what is the most relevant for the addressee's purpose.
(16)There must be something the author of the letter wishes to communicate without stating it in the letter.

That is, behavioral implicatures require understanding the speaker's motives, as well as making assumptions about the addressee's beliefs. This entails that that the derivation of behavioral implicatures is underpinned by at least second-order Theory of Mind.

The same holds for irony; what the speaker intends to communicate through an ironical utterance is inherently different from the primary content. In order to grasp irony it is therefore necessary to make hypotheses about what the speaker believes, as well as about her assumptions about her addressee's beliefs (e.g., Bryant, 2012). For instance, to understand that the speaker of (17) actually hated the movie, the interpreter needs to assume not only that the speaker did not like the movie, but also that the speaker assumes that it is mutually obvious to her and to her addressee that she did not like the movie.

(17) This is the best movie I ever saw.

### 2.3. Pragmatic processing with no theory of mind

At this stage, it becomes natural to question the Relevance theoretic assumption that the output of any type of pragmatic processing consists in a representation of complex communicative intentions. Recall that while Relevance theorists hold that the output of pragmatic processing is always a representation of the speaker's informative intention, they admit different stages of interpretative complexity. Following Sperber's strategy of Naive Optimism, the interpreter may just choose, among different interpretations activated in the context, the one that is the most accessible from his point of view. This interpretative strategy perfectly suits the derivation of material implicatures; as we just saw, these do not require any explicit representation of speaker's mental states. While Jary (2013) holds that Naive Optimism relies on non-mentalistic processes to reach speaker's informative intention or communicative goal, there is no reason why the resulting secondary meaning should necessarily be embedded within a meta-representationally complex attribution of informative intentions to the speaker^1^. The output of the interpretation of *I have already had breakfast* may just be a doxastic-type representation of the content [The speaker does not want a coffee and a croissant]. Likewise, the output of the interpretation of the indirect request *It is cold in here* may just be a conative representation of the addressee's closing the window. Context-sensitive, pragmatic processing should then be possible even in the absence of a second-order Theory of Mind. In addition, as also pointed out by Jary, such pragmatic processing need not be entirely egocentric. As mentioned earlier, very young children are sensitive to other people's perspective, which should allow them to inhibit interpretations that are relevant from their own point of view, but incompatible with the speaker's point of view. It is therefore possible to posit an interpretation process which is sensitive to the speaker's beliefs but that does not rely on complex Theory of Mind neither in its functioning nor, *pace* Relevance theory, in its output.

One may then envision a less homogenous picture of pragmatic processing and modify Sperber's (1994) scale of interpretative strategies as follows (see Jary, 2010, p. 186; Kissine, 2013, pp. 78–80):

1. *Egocentric relevance:* does not require any Theory of Mind, and is entirely based on egocentric considerations of accessibility. The output is the representation of a certain content (viz. non-embedded within the representation of the speaker's informative intentions), and is limited to primary meanings, material implicatures and (some) indirect speech acts;
2. *Allocentric relevance:* requires at least implicit first-order Theory of Mind. It is similar to egocentric relevance, but it rules out contents that are incompatible with the speaker's perspective;
3. “*Gricean”, sophisticated interpretation:* becomes available only when (at least) second-order Theory of Mind is operational. This interpretation strategy involves complex inferences about speaker's communicative intentions, and allows the derivation of behavioral implicatures and comprehension of irony.

This three-pronged hierarchy of interpretative strategies renders unnecessary resorting to a specific pragmatic module. In addition, it is fully consistent with what is known about typical and atypical development. I have argued elsewhere that the first kind of interpretative strategy is at work in persons with autism spectrum disorder (ASD, henceforth) and the second in typically developing children below seven (Kissine, 2012, 2013). There is a robust consensus that, despite individual differences, most children and adults with ASD fail to pass first-order Theory of Mind tasks (e.g., Happé, 1995; Yirmiya et al., 1998; Baron-Cohen, 2000). However, impairment on first-order Theory of Mind does not necessarily prevent people with ASD from using pragmatic processing of the first kind, based on egocentric relevance (Kissine, 2012, 2013). True, there is a broad consensus that individuals with ASD struggle with “social” or “inter-subjective” dimensions of language use. For instance, they often fail to produce informative, new and relevant conversational contributions, to respond on topic and to detect conversational “faux-pas” (e.g., Eales, 1993; Surian et al., 1996; Capps et al., 1998; Surian and Leslie, 1999; Kaland et al., 2002, 2011; Ziatas et al., 2003). However, recent research also shows that persons with ASD are capable to understand metaphors, scalar implicatures (such as non-logical readings of *some*) and even indirect requests (Norbury, 2005; Pijnacker et al., 2009; Chevallier et al., 2010; Gernsbacher and Pripas-Kapit, 2012; Kissine et al., 2012, 2015)^2^. Such a selective pragmatic profile is difficult to explain on a modular theory of pragmatics. By contrast, it makes sense once one admits the existence of egocentric pragmatic processing.

Traditional, verbally demanding versions of first-order Theory of Mind tasks also prove difficult for typically-developing children below the age of four (e.g., Wellman et al., 2001). However, there is evidence that implicit understanding of other people's beliefs is present in typical development as early as at fifteen months (Onishi and Baillargeon, 2005; Southgate et al., 2007; Baillargeon et al., 2010)^3^. Accordingly, typically developing children below four display awareness of their interlocutor's perspective and are already apt conversationalists (see above). That is, typically developing children can exhibit the second, allocentric type of interpretation.

However, until second-order Theory of Mind is mature, roughly around the age of seven, children have difficulties in understanding irony (e.g., Winner and Leekam, 1991; Filippova and Astington, 2008), and do not reach the third, Gricean stage. (Note that on the Sperber and Wilson's idea of a pragmatic module, whose functioning and maturation are independent from Theory of Mind, it is unclear why reaching the developmental stage required for understanding irony should be concomitant with the development of second-order Theory of Mind.)

### 2.4. Interim summary

The foregoing discussion of Relevance theory may be summarized in two general points. First, the steps leading to context-dependent, pragmatic interpretations do not necessarily involve assumptions about the speaker's mental states, but may be based exclusively on contextual accessibility considerations. Second, there are good empirical reasons for believing that context-dependent interpretation of linguistic meaning does not always result in hypotheses about the speaker's complex communicative intentions. In some cases, the interpretation output will consist only in a content that is relevant from the interpreter's own egocentric point of view; in some others, the output will be a content relevant from the speaker's perspective, but without necessarily involving the attribution of complex, multilayered communicative intentions to the speaker.

## 3. Recanati: primary vs. secondary pragmatic processes

Recanati's (2004) two-tiered theory of pragmatic processing is a major contender to the monolithic, modular versions of Relevance theory, discussed in the previous section. The gist of his position is to posit two distinct types—*primary* vs. *secondary*—of pragmatic processing, which differ both in workings and in terms of the output they yield. As his two-pronged theory pragmatic processing does not necessarily rely on assumptions about speaker's communicative intentions, the division of pragmatic labor posited by Recanati seems to be in a better position than Relevance theory to accommodate the empirical data mentioned above. However, on closer inspection the distinction he proposes, be it in terms of internal pragmatic process functioning or output, is not entirely straightforward.

### 3.1. Process workings

Recanati's *secondary pragmatic processes* are Gricean inferences, based on hypotheses about the speaker's intentions. According to him, such secondary pragmatic processes are reserved for the derivation of what has been called here secondary meanings, viz. of contents different from the utterance literal content. By contrast, context-dependent derivation of primary meanings, in Recanati's model, is handled by *primary pragmatic processes*, which operate locally on the linguistic structure of the utterance.

Primary pragmatic processes are determined by accessibility considerations, and do not rely on Theory of Mind. Lexical items give rise to concurrent activation of multiple semantic values. Primary pragmatic processes consist in selecting the most accessible among these values, to subsequently enter within the compositional computation of primary meanings. Primary meanings are thus gradually built as lexical items undergo context-dependent “saturation” (e.g., indexicals such as *she* or demonstratives such as *this* are assigned a referent), “enrichement” (e.g., *and* in *Peter left Mary and she started to drink* is interpreted as *and then, as a result*,), “loosening” (e.g., the meaning of *swallow* in *The ATM swallowed my credit card* is relaxed to apply to non-living organisms), or “free transfer” (e.g., *parked* associated with the speaker and not her car in *I'm parked in the back*).

Now, as discussed in the previous section, it makes sense to assume that some accessibility-based, primary pragmatic processes are sensitive to the speaker perspective, without involving complex mind-reading processes. Admitting that accessibility-based primary pragmatic processing may be allocentric remains compatible with the “Availability criterion” Recanati uses to draw a line between primary and secondary processes. The outputs of primary pragmatic processes, viz. primary meanings, can be made available to conscious introspection, as, for instance, in case of explicit truth-evaluation. Given a (declarative) sentence and a context of utterance, our judgements of truth and falsity, claims Recanati, bear on the contents yielded by primary pragmatic processes. The unfolding of primary pragmatic processes, however, is irreducibly unconscious: interpreters are not conscious of the steps that lead them from linguistic form to pragmatically enriched, primary meanings. Secondary pragmatic processes, by contrast, build on primary meanings, and may thus take the form of a genuine propositional reasoning. Consequently they should be entirely available to conscious introspection. For instance, the derivation of irony may be made available to the interpreter consciousness as a series of inferential steps. In other words, secondary pragmatic processes consist in—or, at least, can be reconstructed as—a sequence of (non-monotonic) inferential steps about the speaker's beliefs and intentions.

### 3.2. Types of processes vs. types of meanings

A consequence of the way Recanati defines primary and secondary pragmatic processes is that in his theory the selection of the type of pragmatic processing—primary or secondary—is entirely determined by the interpretation input. While primary processes operate on lexical items, secondary pragmatic processes are intrinsically “post-propositional”; they consist in an inference from primary to secondary meanings. It thus seems that any pragmatic interpretation that does not directly build on the utterance linguistic structure should only be derivable, in Recanati's theory, through Gricean inferences about speaker's intentions. This is problematic. Implicatures and indirect speech acts are derived, according to Recanati, from primary meanings. However, as we saw in the previous section, material implicatures and indirect speech acts—which are secondary meanings—may be derived with no appeal to the reconstruction of speaker's communicative intentions.

To be fair, it is not obvious whether, for Recanati, the conceptual precedence of primary over secondary pragmatic processes extends to psychological processing. He does go at great lengths to argue that implicature derivation is not necessarily handled by conscious and voluntary inferences about the speaker's intentions. Yet, such pragmatic processing is still secondary, according to him, because the inference from primary meaning to the implicature is available, *ex post facto*, to the interpreter's consciousness (Recanati, 2004, pp. 46–50, 70–71). Under one interpretation of this claim, secondary pragmatic processes may occur both at unconscious and conscious levels, but still differ from primary ones in terms of their workings. Derivation of secondary meanings should then always presuppose a complex Theory of Mind, which would make developmental data discussed above as problematic for Recanati as it is for Sperber and Wilson. Recall, for exemple, that children with ASD (Kissine et al., 2012, 2015), as well as typically developing toddlers (Reeder, 1978; Shatz, 1978; Schulze and Tomasello, 2015), understand indirect requests. Another reading of Recanati's theory, more in line with the view argued for at the end of the previous section, is that some secondary meanings, such as material implicatures and indirect speech acts, may be derived through either primary or secondary pragmatic processes. This reinterpretation of Recanati's theory entails that types of pragmatic processing do not necessarily correlate with types (primary vs. secondary) of meanings. While this is the view I wish to defend, it is important to emphasize that the challenge now becomes to explain what drives the selection of the pragmatic process type.

### 3.3. Interim summary

At this stage, we reach a rather complex picture. In agreement with Jary and Recanati, it makes sense to posit that some context-dependent, pragmatic processes do not involve Theory of Mind, but are accessibility-based. However, types of pragmatic processes do not correlate with types of meanings, as some secondary meanings (material implicatures and indirect speech acts) may be derived using contextual accessibility alone, without involving Theory of Mind. In addition, while the output of some pragmatic processing consists in attributing complex communicative intentions to the speaker, contextual interpretation of linguistic meaning may also yield representations of the utterance contents without involving any representation of the speaker's mental states. That is, secondary meanings, such as material implicatures or indirect speech acts, need not correspond to a complex meta-representation of speaker's informative intention. In Section 5, I will sketch a proposal where types of pragmatic processes are not determined by types of meanings. The main idea will be that types of meanings (primary or secondary) are recovered relative to contextually determined norms of interpretation, which may, but need not target speaker's communicative intentions, and may be entirely egocentric or partly depend on the speaker's perspective. Pragmatic processes lead to and monitor the construction of utterance contents relative to norms of interpretation.

While this way of thinking about pragmatics may seem quite unusual, it has in fact straightforward parallels in cognitive science. On such a conception, pragmatic processing belongs to the broader category of meta-cognitive processes associated with *epistemic acceptance*. More particularly, the model I will defend has clear parallels with a contextualist view of epistemic acceptance, to this discussion of which I turn now.

## 4. Two kinds of epistemic acceptance

Acceptance refers to a mental action that consists in including a proposition among one's beliefs (hence making it available for subsequent action planning and inference). Thus defined, acceptance includes, for instance, accepting that one's recollection of an event is faithful enough, that a story one hears is truthful or that one's interpretation of a difficult passage in a book is accurate enough.

From an epistemological point of view, there are two, equally plausible, norms for acceptance that a rational system should follow. The first norm for acceptance is set relative to a certain confidence threshold; whenever a rational agent has a certain degree of confidence *n* that a proposition *p* is true—such that, say, 0.5 < *n* ≤ 1—she should accept *p*. The second norm obeys consistency requirements: a rational agent should accept any consequence that follows from a proposition or a conjunction of propositions she previously accepted. However, the conjunction of these two norms begets two notorious paradoxes (Hempel, 1962; Makinson, 1965). The first paradox is standardly illustrated with a lottery example. Imagine a lottery with one winning ticket over a thousand. When one buys a ticket, there is a probability of 0.999 that it will lose. Since 0.999 is a fairly reasonable confidence threshold, the first epistemic norm of acceptance dictates that the buyer should accept that her ticket will lose. Now, every ticket has exactly the same chance to win, and, accordingly, one should accept, about each individual ticket, that it will not win. However, it follows, from the second norm of acceptance, that one should accept that no ticket in the whole lot will win. To see the second paradox imagine a historian that compiles a lifelong work on, say, the reign of Peter the Great. As she writes, she has sufficient confidence for accepting each claim she makes. However, given the breadth of her endeavor it seems that it would be rational for her to accept that, as a whole, her book may contain some inaccuracies. Yet, if the book is taken as the sum of individual claims she accepted on the basis of the first norm, this acceptance should be irrational.

These two infamous paradoxes may be dissolved by acknowledging that norms for epistemic acceptance vary context from context (Kaplan, 1981). In some contexts, it is the proximity to truth that is important—e.g., *How exact is my recollection of a particular utterance?*—and it is the first norm that applies. In some others, it is the internal consistency of a set of propositions that matters—e.g., *How consistent is my recollection of a conversation?*—and it is the second norm that applies. Building on Kaplan's idea, Proust (2013, chapter 8) points out that there are more norms than these two. Acceptance may be guided by adherence to a shared disposition to act in a group; for instance, in conducting peace talks, a proposition ought to be accepted, if, and only if, it is coherent with the team's general plan of negotiation. Or, in writing a novel, the writer will be guided in her acceptance of a proposition by coherence with the fiction background.

The major consequence of this contextualist view of acceptance is that the selection of the acceptance norm, viz. of the kind of acceptance at stake, is independent of the monitoring of success relative to this norm. The selection of this or that norm of acceptance depends on one's appraisal of the environment and practical goals: should I privilege truth, consistency, adequacy with my group plans.…Acceptance of the proposition itself, e.g., its integration within one's beliefs or within a line of argument, proceeds relative to this norm. The adequacy of the process of acceptance relative to the norm is then monitored and controlled at a *metacognitive* level, by specific procedural loops operating on aspects of cognitive processes (Koriat, 2000; Proust, 2013).

It is standard to draw a distinction between types of metacognitive control that can be made available to conscious introspection and those that are best seen as unconscious processes (Koriat, 2000; Shea et al., 2014 ; for a more nuanced view, see Metcalfe and Son, 2012). *Meta-cognitive judgements* may be brought to consciousness and take the form of deliberate inferences about one's beliefs and memories. An instance of a meta-cognitive judgement is making an inference about one's likelihood to provide an answer in a memory task, based, for instance, on the task complexity and previous experience. *Meta-cognitive feelings* also provide feedback in the epistemic domain, but they are difficult to reconstruct in inferential terms. The clearest example of a meta-cognitive feeling is the “tip-of-the-tongue” experience: the subject can more or less accurately assess the likelihood of her recalling an information to which, however, she has no conscious access. Applied to epistemic acceptance, meta-cognitive feelings may provide the subject with an assessment of the adequacy of the output relative to the norm without her having conscious access to the grounds of this normative assessment.

This latter point is consistent with the idea that although metacognition operates on cognitive processes it does not entail meta-representation of mental states. The two main arguments for holding that metacognitive does not require mindreading are: (a) the differential appraisal of one's own and other people's performance on a cognitive task, and (b) the evidenced metacognitive processes in vertebrates that have no Theory of Mind (e.g., Koriat and Goldsmith, 1996; Koriat, 2000; Proust, 2013; Shea et al., 2014).

To sum up, a contextualist theory of epistemic acceptance entails the following three fold procedural distinction:

1. Context-dependent selection of the norm of acceptance;
2. The metacognitive process that monitors and controls acceptance;
3. The resulting acceptance (or integration).

I will now argue that this distinction exactly parallels pragmatic processing of a linguistic utterance. To the context-dependent norms of acceptance correspond context-dependent norms of interpretation; to the metacognitive processes correspond pragmatic processes, and to the result, i.e., to the acceptance itself, correspond the final representation(s) of the utterance content(s).

## 5. Norms of interpretation vs. pragmatic processes

Deriving the meaning of an utterance is an epistemic operation, which terminates with the acceptance of a particular interpretation. Just as one should distinguish between the contextual selection of an acceptance norm and the acceptance process relative to this norm, one should not confuse the norm of an interpretation process with the interpretation process itself. A good way to understand this point is to consider the different ways utterance interpretation may go wrong. The distinction between the selection of the acceptance norm, and acceptance as assessment relative to this norm entails that one may be wrong in two different ways: either by failing to select the contextually appropriate acceptance norm or because of a meta-cognitive failure to assess adequately one's judgement relative to this norm. The same applies to pragmatic processing. Take, as an illustration, irony misunderstandings. There are two ways one can fail to understand irony. One may fail to understand that the speaker is being ironic and stick to the literal interpretation. In this case, the norm of interpretation has not been adequately set relative to the context of conversation. Or, one may understand that the speaker is being ironic but fail to discern what she actually means (realizing one's failure or not). This time the interpretative norm has been adequately set; however, either no interpretation is arrived at (as the interpreter adequately rejects all candidate contents) or the interpretation process delivers a content the interpreter mistakenly accepts as contextually adequate.

As we saw earlier, understanding irony requires grasping speaker's beliefs and intentions; the appropriate contextual norm here is the recovery of the speaker's communicative intentions. Interpreting the utterance relative to this norm thus requires a specific monitoring and control process, which must draw on the attribution of second-order mental states. Once such a complex interpretative norm has been set, the control mechanism that yields awareness of interpretative failure or success is probably an instance of metacognitive judgement, which can be explicitly reconstructed as an inferential explanation (based on standard Gricean considerations of discrepancy between the literal meaning and the context).

Contrasting with irony, the first two interpretative strategies identified at the end of Section 2—egocentric and allocentric relevance—correlate with more modest norms of egocentric relevance, mitigated or not by the integration of the speaker's perspective. Pragmatic processes guided by such less complex interpretative norms rely on contextual accessibility without involving complex mind-reading. That is, they terminate once a sufficiently accessible interpretation has been reached.

It is worth emphasizing that such processes are genuinely context-dependent, and not guided by mere salience. In Recanati's (2004, p. 30) definition, the most accessible meaning of a lexical item “corresponds to the most active interpretation when the interpretation process stabilizes.” Salience of a lexical meaning may be determined by its frequency of use, familiarity to the interpreter or prototypicality. Lexical meanings are activated according to their relative salience independently of and in parallel to contextual factors, which means that in contexts that favor non-salient meanings of a lexeme, its salient, but contextually inadequate meanings are still activated (see Giora, 2003). For instance, the most salient (frequent) meaning of *bulb* is [light bulb]. Peleg et al. (2001) found that this meaning is activated in (18), even though it is contextually inappropriate.

(18) The gardener dug a hole. *The bulb* was inserted

Accordingly, even when the interpretation norm is simple egocentric relevance, metacognitive control will be intrinsically context-sensitive. For instance, it is very plausible that the contextually adequate interpretation of *bulb* in (20) may be reaching using an entirely egocentric interpretative norm. However, this interpretation process is pragmatic as it involves inhibiting the salient, but contextually inadequate meaning *light bulb*, which was automatically activated.

In line with Recanati's theory, accessibility-based processes probably remain out of conscious reach of the interpreters. While the progression of Gricean inferential processes, such as irony derivation, is controlled by metacognitive judgements, control of unconscious pragmatic processes is more likely to correspond to a metacognitive feeling. To be sure, this idea needs extensive empirical confirmation. However, it is intuitively plausible that garden-path interpretations are accompanied by a distinctive feeling “of something being wrong.” As we just saw, the most salient meaning of *bulb* is “light bulb”; as a result, when the interpretation of (19) reaches the end of the sentence, backtracking is likely to occur.

(19) The bulbs John stored in his closet have flowered.

It seem plausible that this backtracking is accompanied by a metacognitive feeling of interpretative failure. If so, there is a similarity between the meta-cognitive feelings associated with non-Gricean pragmatic processes and the tip-of-tongue phenomenon: in both cases, the metacognitive feeling provides conscious feedback on an unconscious process.

Independently of the validity of the contrast between metacognitive judgements and feelings, be it in pragmatics or more generally, the parallel I am drawing between metacognitive control of epistemic acceptance and pragmatic processing provides a fresh conceptual framework for thinking about the ways context determines utterance interpretation. Context plays two distinct roles, which should not be confused. First, context is required to set up an interpretation norm. In some contexts, this norm will be complete recovery of the speaker's communicative intentions (for instance, the conversation is full of innuendo or the speaker is being clearly sarcastic). In some other contexts, the norm is the interpretation that is the most relevant given the speaker's perspective. And in still some other contexts, simple egocentric relevance is sufficient. Contextual selection of appropriate interpretative norms is largely independent of the linguistic input; drawing on world-knowledge and interactional experiences, it consists in the assessment of the frame of interaction^4^.

Second, the success of interpretative processes must be monitored and controlled relative to this norm. That is, pragmatic processes involve contextual selection among activated meanings and assessment of the unfolding interpretation relative to the interpretative norm. The input of the pragmatic interpretative processes is restricted to linguistic, or at least, communicative stimuli. However, the kind of contextual resources required for pragmatic processing—and in particular, the extent to which it draws on Theory of Mind—depends on the interpretative norm it is geared to. In sum, the selection of interpretative norms is *context-driven*, while pragmatic processing is *context-senstive* but driven by the selected interpretative norm.

The model I propose is summarized in Table 1. Its most important feature is that the typology of pragmatic processes is not determined by a hierarchy of meanings. Whether pragmatic processing involves Theory of Mind or not depends on the kind of interpretative norm that has been contextually selected. The crucial empirical prediction that follows is that some kinds of meaning may be derived through different types of pragmatic processing. This is consistent with the fact that, as we saw above, material implicatures or indirect speech acts may sometimes be interpreted in an entirely egocentric way.

**Table 1:** **Meanings vs. pragmatic processes vs. interpretation norms**.

**Types of meanings**		**Types of processing**	**Interpretation norms**
Primary meanings		Egocentric or allocentric accessibility	Egocentric or allocentric relevance
Secondary meanings	Material implicatures; indirect speech acts		
	Behavioral implicatures; irony	Gricean	Speaker's motives and intentions

Another straightforward prediction of this model is that the repertoire of available interpretative norms varies across interpreters. Drawing again a parallel with epistemic acceptance, acquiring some epistemic norms—e.g., logical consistency—requires considerable cognitive maturation, and emerge late in ontogenesis. Likewise, the interpretative norm consisting in the full recovery of the speaker's communicative intentions should not emerge until second-order Theory of Mind is mature. Some secondary meanings, such as irony, cannot be derived in the absence of such complex interpretative norms; some others, however, such as material implicatures and indirect speech acts, may be reached through less complex processing. This is why appropriate indirect request understanding has been observed in typically developing toddlers (Shatz, 1978; Reeder, 1978; Schulze and Tomasello, 2015) and children with ASD (Kissine et al., 2012, 2015), two population with no complex Theory of Mind^5^.

This last point should not be taken as implying that once a more complex interpretative norm is operational it overwrites less complex norms, which were available earlier on; rather, pragmatic development enriches the repertoire of interpretative norms, which all remain available to the interpreter. It is plausible to assume that when a less demanding norm, such as egocentric relevance, appears to be suitable, it will be privileged over the more complex, Gricean norm. In many contexts competent, adults interpreters limit themselves to such an egocentric interpretation^6^. This is also consistent with the idea that, in most situations, interpreters automatically integrate the utterance content within their beliefs. On the model defended here, interpretation outputs are not always embedded within complex meta-representations of the speaker's communicative intentions (see Kissine, 2013, pp. 80–101, Kissine and Klein, 2013). While such meta-representational outputs probably form a barrier against automatic integration (cf. Sperber et al., 2010), they will not emerge in contexts where interpretation is geared toward a less complex interpretation norm.

A connected prediction is that differential processing of the same stimuli may be evidenced in experimental paradigms that make salient different interpretation norms to participants^7^. Therefore, great care must be paid, in the interpretation of experimental results, not to confuse the type of meaning supposed to be illustrated by the stimuli and the actual processing that took place in the participants' minds.

As a brief example of this last point, take irony comprehension in autism. As already mentioned, there is a widespread consensus that irony comprehension requires second-order Theory of Mind (e.g., Bryant, 2012). It is therefore expected that persons with ASD who do not have second-order Theory of Mind fail to understand irony (Happé, 1993; Leekam and Prior, 1994; Martin and McDonald, 2004). It may seem surprising, then, that in Chevallier et al. (2011) and Colich et al. (2012) participants with ASD correctly discriminate between “ironical” and “literal” interpretations. However, that the task in these two studies consisted in choosing between two responses, literal vs. ironic. Furthermore, unlike their literal counterpart, ironic stimuli were incongruent with the preceding context and characterized by a marked intonation. In real-life situations, the interpretative norm associated with ironical interpretation is the recovery of the speaker's intentions. However, in the experimental studies under discussion, the more modest norm, consisting in rejecting the literal interpretation, sufficed to provide the correct response (a consequence acknowledged by Colich et al., 2012). Pragmatic processing guided by such a norm may remain entirely egocentric and accessibility-based in its workings.

## 6. Conclusion

This paper urges a change of perspective on pragmatic processing by distinguishing contextually-dependent selection of interpretative norms and context-sensitive pragmatic processing. A crucial feature of this proposal is that types of processing (accessibility based vs. Gricean) do not correlate with types of meaning (primary vs. secondary). At this stage, the model remains largely speculative, and many details need to be filled in. For instance, while I focused on three interpretative norms, inspired by Sperber (1994) and Jary (2010, pp. 185–187), there may be more. In addition, links between general metacognitive control and pragmatic performance should be empirically investigated. However, the model proposed allows a better integration of experimental data on pragmatic processing in early typical and atypical development. Furthermore, it contributes to building a research framework within which the interpretation of experimental results is sensitive to the interpretative norms participants are likely to select.

## Funding

This research is part of a research project funded by the F.R.S.-FNRS Research Incentive Grant F.4502.15 and the Fédération Wallonie-Bruxelles ARC-Consolidator Grant “Context in Autism.”

### Conflict of interest statement

The author declares that the research was conducted in the absence of any commercial or financial relationships that could be construed as a potential conflict of interest.

## Footnotes

^1^But see Jary (2010, pp. 183–185).

^2^To be more precise, metaphor comprehension is impaired in many individuals with autism, but this impairment seems to be caused, to a large extent, by reduced receptive vocabulary, and not by Theory of Mind deficits (e.g., Norbury, 2005).

^3^Individuals with ASD, by contrast, do no seem to deploy such implicit belief understanding (Senju et al., 2010).

^4^Of course, in some cases the linguistic input may trigger the switch from an egocentric to a more complex Gricean interpretative norm. In particular, search for ironic interpretation may be primed by prosody or discourse context (e.g., Kowatch et al., 2013; Spotorno and Noveck, 2014). More often, however, ironic interpretation will be triggered because interpretation driven by a more modest norm fails to deliver any plausible output.

^5^See also Spotorno and Noveck (2014) on individual differences in strategies used for irony detection.

^6^This assumption has interesting parallels with the “Good enough” approach, according to which syntactic and semantic processing remains shallow whenever a detailed interpretation is not required by the task at hand (Ferreira and Patson, 2007).

^7^In fact, this point is consonant with the well-established finding that some cognitive biaises may be reduced by preventing participants from reading experimenter's intentions within experimental instructions (e.g., Wright and Wells, 1988; Schwarz et al., 1991).

## References

[B1] Bach K. (1994). Semantic slack. What is said and more, in Foundations of Speech Act Theory. Philosophical and Linguistic Perspectives, ed Tsohatzidis S. (London: Routledge), 267–291.

[B2] Baillargeon R. Scott R. M. He Z. J. (2010). False-belief understanding in infants. Trends Cogn. Sci. 14, 110–118. 10.1016/j.tics.2009.12.006 20106714 PMC2930901

[B3] Baron-Cohen S. (2000). Theory of mind and autism: a fifteen year review, in Understanding Other Minds. Perspectives from Developmental Cognitive Science, 2nd Edn., eds Baron-Cohen S. Tager-Flusberg H. Cohen D. J. (Oxford: Oxford University Press), 3–20.

[B4] Bryant G. A. (2012). Is verbal irony special? Lang. Linguist. Compass 6, 673–685. 10.1002/lnc3.364

[B5] Capps L. Kehres J. Sigman M. (1998). Conversational abilities among children with autism and children with developmental delays. Autism 2, 325–344.

[B6] Carston R. (2002). Thoughts and Utterances. The Pragmatics of Explicit Communication. Oxford: Blackwell.

[B7] Chevallier C. Noveck I. Happé F. G. E. Wilson D. (2011). What's in a voice? Prosody as a test case for the Theory of Mind account of autism. Neuropsychologia 49, 507–517. 10.1016/j.neuropsychologia.2010.11.042 21134386

[B8] Chevallier C. Wilson D. Happé F. G. E. Noveck I. (2010). Scalar inferences in autism spectrum disorders. J. Autism Dev. Disord. 40, 1104–1017. 10.1007/s10803-010-0960-8 20143144

[B9] Colich N. L. Wang A.-T. Rudie J. D. Hernandez L. M. Bookheimer S. Y. Dapretto M. (2012). Atypical neural processing of ironic and sincere remarks in children and adolescents with Autism Spectrum Disorders. Metaphor Symbol 27, 70–92. 10.1080/10926488.2012.638856 24497750 PMC3909704

[B10] Eales M. J. (1993). Pragmatic impairments in adults with childhood diagnoses of autism or developmental receptive language disorder. J. Autism Dev. Disord. 23, 593–617. 8106302 10.1007/BF01046104

[B11] Ferreira F. Patson N. D. (2007). The “Good Enough” approach to language comprehension. Lang. Linguist. Compass 1, 71–83. 10.1111/j.1749-818X.2007.00007.x

[B12] Filippova E. Astington J. W. (2008). Further development in social reasoning revealed in discourse irony understanding. Child Dev. 79, 126–138. 10.1111/j.1467-8624.2007.01115.x 18269513

[B13] Gernsbacher M. A. Pripas-Kapit S. R. (2012). Who's missing the point? A commentary on claims that autistic persons have a specific deficit in figurative language comprehension. Metaphor Symbol 27, 93–105. 10.1080/10926488.2012.656255 25339845 PMC4203428

[B14] Geurts B. (2010). Quantity Implicatures. Cambridge: Cambridge University Press.

[B15] Giora R. (2003). On our Mind. Salience, Context, and Figurative Language. Oxford: Oxford University Press.

[B16] Grice H. P. (1957). Meaning. Philos. Rev. 66, 377–388. 26564604

[B17] Grice H. P. (1975). Logic and conversation, in Syntax and Semantics 3: Speech Acts, eds Cole P. Morgan J. L. (New York, NY: Academic Press), 41–58.

[B18] Grosse G. Moll H. Tomasello M. (2010). 21-month-old understand the cooperative logic of requests. J. Pragmat. 42, 3377–3383. 10.1016/j.pragma.2010.05.005

[B19] Happé F. G. E. (1993). Communicative competence and Theory of Mind in autism. A test of relevance theory. Cognition 48, 101–119. 8243028 10.1016/0010-0277(93)90026-r

[B20] Happé F. G. E. (1995). The role of age and verbal ability in the Theory of Mind task performance of subjects with autism. Child Dev. 66, 843–855. 7789204

[B21] Hempel C. G. (1962). Deductive-nomological vs. statistical explanation. Minnesota Stud. Philos. Sci. 3, 98–169.

[B22] Jary M. (2010). Assertion. London: Palgrave.

[B23] Jary M. (2013). Two types of implicature: material and behavioural. Mind Lang. 28, 638–660. 10.1111/mila.12037

[B25] Kaland N. Moller-Nielsen A. Callesen K. Mortensen E. L. Gottlieb D. Smith L. (2002). A new ‘advanced’ test of theory of mind: evidence from children and adolescents with Asperger syndrome. J. Child Psychol. Psychiatry 43, 517–528. 10.1111/1469-7610.00042 12030597

[B26] Kaland N. Mortensen E. L. Smith L. (2011). Social communication impairments in children and adolescents with Asperger syndrome: slow response time and the impact of prompting. Res. Autism Spectrum Disord. 5, 1129–1137. 10.1016/j.rasd.2010.12.009

[B27] Kaplan M. (1981). Rational acceptance. Philos. Stud. 40, 129–145.

[B28] Kissine M. (2012). Pragmatics, cognitive flexibility and Autism Spectrum Disorders. Mind Lang. 28, 1–28. 10.1111/j.1468-0017.2011.01433.x

[B29] Kissine M. (2013). From Utterances to Speech Acts. Cambridge: Cambridge University Press.

[B30] Kissine M. De Brabanter P. Leybaert J. (2012). The interpretation of requests in children with autism: the effect of the sentence-type. Autism 16, 523–532. 10.1177/1362361311406296 22399448

[B31] Kissine M. Cano-Chervel J. Carlier S. De Brabanter P. Ducenne L. Pairon M.-C.. (2015). Children with autism understand indirect speech acts: evidence from a semi-structured act-out task. PLoS ONE 10:e0142191. 10.1371/journal.pone.0142191 26551648 PMC4638355

[B32] Kissine M. Klein O. (2013). Models of communication, epistemic trust and epistemic vigilance, in Social Cognition and Communication, eds Forgas J. P. Vincze O. Lázló J. (New York, NY: Psychology Press), 139–154.

[B33] Koriat A. (2000). The feeling of knowing: some metatheoretical implications for consciousness and control. Consciousness Cogn. 9, 149–171. 10.1006/ccog.2000.0433 10924234

[B34] Koriat A. Goldsmith M. (1996). Monitoring and control processes in the strategic regulation of memory accuracy. Psychol. Rev. 103, 490–517. 8759045 10.1037/0033-295x.103.3.490

[B35] Kowatch K. Whalen J. M. Pexman P. M. (2013). Irony comprehension in action: a new test of processing verbal irony. Discourse Process. 50, 301–315. 10.1080/0163853X.2013.799934

[B36] Leekam S. R. Prior M. (1994). Can autistic children distinguish lies from jokes? A second look at second-order belief attribution. J. Child Psychol. Psychiatry 35, 901–915. 7962247 10.1111/j.1469-7610.1994.tb02301.x

[B37] Liszkowski U. Carpenter M. Striano T. Tomasello M. (2006). 12- and 18-month-olds point ot provide information for others. J. Cogn. Dev. 7, 173–187. 10.1207/s15327647jcd0702_2

[B38] Liszkowski U. Carpenter M. Tomasello M. (2008). Twelve-month-olds communicate helpfully and appropriately for knowledgeable and ignorant partners. Cognition 108, 72–739. 10.1016/j.cognition.2008.06.013 18721918

[B39] Makinson D. C. (1965). The paradox of the preface. Analysis 25, 205–207. 10.1093/analys/25.6.205

[B40] Martin I. McDonald S. (2004). An exploration of causes of non-literal language problems in individuals with Asperger Syndrome. J. Autism Dev. Disord. 34, 311–328. 10.1023/B:JADD.0000029553.52889.15 15264499

[B41] Matthews D. Lieven E. Tomasello M. (2010). What's in a manner of speaking? Children's sensitivity to partner-specific referential precedents. Dev. Psychol. 46, 749–760. 10.1037/a0019657 20604599

[B42] Metcalfe J. Son L. (2012). Anoetic, noetic, and autonoetic metacognition, in The Foundations of Metacognition, eds Brenan M. Brandl J. Perner J. Proust J. (Oxford: Oxford University Press), 289–301.

[B43] Norbury C. F. (2005). The relationship between theory of mind and metaphor: evidence from children with language impairment and autistic spectrum disorder. Br. J. Dev. Psychol. 23, 383–399. 10.1348/026151005X26732

[B44] Noveck I. (2001). When children are more logical than adults: experimental investigations of scalar implicature. Cognition 78, 165–188. 10.1016/S0010-0277(00)00114-1 11074249

[B45] Onishi K. Baillargeon R. (2005). Do 15-month-old infants understand false beliefs? Science 308, 255–258. 10.1126/science.1107621 15821091 PMC3357322

[B46] Peleg O. Giora R. Fein O. (2001). Salience and context effects: two are better than one. Metaphor Symbol 16, 173–192. 10.1080/10926488.2001.9678894

[B47] Perner J. Wimmer E. (1985). “John thinks that Mary thinks that…”. Attribution of second-order belief by 5- to 10-year-old children, J. Child Exp. Psychol. 39, 437–471.

[B48] Pijnacker J. Hagoort P. Buitelaar J. Teunisse J.-P. Geurts B. (2009). Pragmatic inferences in high-functionning adults with autism and Asperger syndrome. J. Autism Dev. Disord. 39, 607–618. 10.1007/s10803-008-0661-8 19052858

[B49] Proust J. (2013). The Philosophy of Metacognition: Mental Agency and Self-awareness. Oxford: Oxford University Press.

[B50] Recanati F. (2004). Literal Meaning. Cambridge: Cambridge University Press.

[B51] Reeder K. (1978). The emergence of illocutionary skills. J. Child Lang. 7, 13–28. 7372729 10.1017/s0305000900007005

[B52] Schwarz N. Strack F. Hilton D. J. Naderer G. (1991). Base rates, representativeness, and the logic of conversation: the contextual relevance of “irrelevant” information. Soc. Cogn. 9, 67–84.

[B53] Schulze C. Grassmann S. Tomasello M. (2013). 3-year-old children make relevance inferences in indirect verbal communication. Child Dev. 84, 2079–2093. 10.1111/cdev.12093 23550944

[B54] Schulze C. Tomasello M. (2015). 18-month-olds comprehend indirect communicative acts. Cognition 136, 91–98. 10.1016/j.cognition.2014.11.036 25497519

[B55] Senju A. Southgate V. Miura Y. Matsui T. Hasegawa T. Tojo Y. (2010). Absence of spontaneous action anticipation by false belief attribution in children with Autism Spectrum Disorders. Dev. Psychopathol. 22, 353–360. 10.1017/S0954579410000106 20423546

[B56] Shatz M. (1978). On the development of communicative understandings: an early strategy for interpreting and responding to messages. Cogn. Psychol. 10, 271–301.

[B57] Shea N. Boldt A. Bang D. Yeung N. Heyes C. Frith C. D. (2014). Supra-personal cognitive control and metacognition. Trends Cogn. Sci. 18, 186–193. 10.1016/j.tics.2014.01.006 24582436 PMC3989995

[B58] Shwe H. Markman E. (1997). Young children's appreciation of the mental impact of their communicative signals. Dev. Psychol. 33, 630–636. 9232378 10.1037//0012-1649.33.4.630

[B59] Southgate V. Senju A. Csibra G. (2007). Action anticipation through attribution of false belief by 2-year-olds. Psychol. Sci. 18, 587–592. 10.1111/j.1467-9280.2007.01944.x 17614866

[B60] Sperber D. (1994). Understanding verbal understanding, in What is Intelligence? ed Khalfa J. (Cambridge: Cambridge University Press), 179–198.

[B61] Sperber D. Clément F. Heintz C. Mascaro O. Mercier H. Origgi G. (2010). Epistemic vigilance. Mind Lang. 25, 359–393. 10.1111/j.1468-0017.2010.01394.x

[B62] Sperber D. Wilson D. (1995). Relevance: Communication and Cognition, 2nd Edn. Oxford: Blackwell.

[B63] Sperber D. Wilson D. (2002). Pragmatics, modularity and mind-reading. Mind Lang. 17, 3–23. 10.1111/1468-0017.00186

[B64] Spotorno N. Noveck I. (2014). When is irony effortful? J. Exp. Psychol. Gen. 143, 1649–1665. 10.1111/1468-0017.00186 24773194

[B65] Surian L. Baron-Cohen S. Van der Lely H. (1996). Are children with autism deaf to Gricean Maxims? Cogn. Neuropsychiatry 1, 55–71. 16571474 10.1080/135468096396703

[B66] Surian L. Leslie A. M. (1999). Competence and performance in false belief understanding: a comparison of autistic and normal 3-year-old children. Br. J. Dev. Psychol. 17, 141–155.

[B67] Wellman H. M. Cross D. Watson J. (2001). Meta-analysis of theory-of-mind development: the truth about false belief. Child Dev. 72, 655–684. 10.1111/1467-8624.00304 11405571

[B68] Winner E. Leekam S. R. (1991). Distinguishing irony from deception: understanding the speaker's second-order intention. Br. J. Dev. Psychol. 9, 257–270.

[B69] Wright E. F. Wells G. L. (1988). Is the attitude-attribution paradigm suitable for investigating the dispositional bias? Pers. Soc. Psychol. Bull. 14, 183–190. 10.1177/0146167288141018 30045458

[B70] Yirmiya N. Erel O. Shaked M. Solomonica-Levi D. (1998). Meta-analyses comparing theory of mind abilities of individuals with autism, individuals with mental retardation, and normally developing individuals. Psychol. Bull. 124, 283–307. 9849110 10.1037/0033-2909.124.3.283

[B71] Ziatas K. Durkin K. Pratt C. (2003). Differences in assertive speech acts produced by children with autism, Asperger syndrome, specific language impairment, and normal development. Dev. Psychopathol. 15, 73–94. 10.1017/S0954579403000051 12848436

